# Evidence of past dental visits and incidence of head and neck cancers: a systematic review and meta-analysis

**DOI:** 10.1186/s13643-019-0949-0

**Published:** 2019-02-04

**Authors:** Bhawna Gupta, Narinder Kumar, Newell W. Johnson

**Affiliations:** 1 Torrens University, Adelaide, South Australia 5000 Australia; 2 Department of Orthopaedics, Base Hospital, Lucknow, India; 3 Menzies Health Institute Queensland and School of Dentistry and Oral Health, Griffith University, Gold Coast, Australia

**Keywords:** Oral cancers, Head and neck cancers, Upper aerodigestive tract cancers, Dental visits, Dental check-ups, Systematic review and meta-analysis

## Abstract

**Background:**

Regular/frequent dental visits, at least annually, can aid in reducing the public health burden of head and neck cancers (HNCs) by facilitating earlier detection of the disease. The aim of this study was to conduct a quantitative assessment of any independent association between past dental visits/check-ups and incidence of cancers of HN/upper aerodigestive tract (UADT) and oral cavity worldwide.

**Methods:**

PubMed, CINAHL, and Cochrane databases were searched for all observational studies published until August 2017 in any language that assessed an association of past dental visits/dental check-ups among the incident cases of HNC/UADT cancers. Screening and quality assessment of the articles was performed by two independent reviewers. Three different meta-analyses were conducted: two based on the incident cancer reported in the studies (HNCs/cancers of UADT and oral cavity); another included all studies irrespective of the type of cancer reported with the frequency of past dental visits as subgroups.

**Results:**

Searches retrieved 3164 titles: after removing duplicates, 1377 remained. Of these, 62 were reviewed in full, but only 38 were eligible for inclusion. Under the random effects model, odds of past never/irregular/not frequent dental visits were greater in HNC cases and oral cancer cases as compared to the hospital-based/population-based controls [HNCs-unadjusted odds ratio (OR) 2.24; 95% confidence interval (CI) 1.89 to 2.65) and (oral cancers—OR 1.93; 95% CI 1.47 to 2.52]. Similar results were observed for all cancers with frequency of past dental visits as subgroup analysis (OR 2.01; 95% CI 1.76 to 2.30). Meta-regression findings indicate that none of the subgroup influenced the effect estimates for incidence of cancers. There was no publication bias in our study.

**Conclusion:**

This systematic review and meta-analysis indicates that individuals with never/irregular/not frequent dental visits are more likely to be incident cases of HNCs/UADT cancers.

## Introduction

Routine/frequent dental visits can aid in detection of head and neck cancers (HNCs) at an early stage [1–4]. More than 90% of HNCs are squamous cell carcinomas that arise from the mucosal lining of the upper aerodigestive tract (UADT). As defined by the World Health Organization International Classification of Disease (ICD-10 version 2015), cancers of oral cavity (C00-06), oropharynx (C010), hypopharynx (C13), larynx (C32), and esophagus (C15) are collectively known as UADT cancers [5]. Although oral cancers (OCs: the commonest site of HNC worldwide) can be detected early with a simple oral examination as compared to cancers that involve more elaborate screening tests (i.e., breast, prostate, and colon), the rate of early diagnosis of OCs has not improved over time with advanced disease at presentation ranging from 27 to 77% across the globe [6].

In high-risk populations, some OCs may be preventable through identification of oral potentially malignant disorders by general dental practitioners facilitating diagnosis at an early stage, thus initiating the first line of treatment, enabling better treatment outcomes, and lowering the cost of care [7]. Diagnosis of cancer at an early stage can thereby improve survival rates in addition to obtaining better function and esthetics for patients. Visual examination and palpation are the standard mode of OCs screening in wide-spread use. Such opportunistic screening for OCs in both high- and low-risk patients during routine dental check-ups is more likely to be cost-effective in comparison to systematic population-based screening programmes [8, 9]. Dentists may be particularly well-suited to perform such oral cavity examinations due to their scientific training with the oral anatomy and professional access to the oral cavity [10]. However, OCs are often not conspicuous and thus early detection requires great skill and care, necessitating an informed pool of dentists to conduct thorough examinations on a regular basis among high-risk patients [11–13].

The present systematic review and meta-analysis aims to critically appraise data from comparable observational studies published in any part of the world, leading to a quantitative summary of the scientific evidence of past dental visits versus never dental visits and its association with the incidence of cancers of HN/UADT and oral cavity worldwide. To the best of our knowledge, there is no previously published systematic review and meta-analysis on this topic.

## Materials and methods

We followed the Preferred Reporting Items for Systematic Review and Meta-Analysis (PRISMA) strategy [14]. We have used the critical appraisal skills programme checklist to systematically assess the relevance and results of published papers (https://casp-uk.net/). Case-control, prospective and retrospective cohort, cross-sectional, and screening studies that assessed an association between past dental visits/dental check-ups among the incident cases of HNC and UADT cancers were considered for inclusion. This meta-analysis is based on MOOSE guidelines: Meta-analysis of Observational Studies in Epidemiology [15].

### Literature search strategy

We identified all the published studies using an extensive search of the PubMed, CINAHL, and the Cochrane database from the inception of relevant database until August 2017. The following search terms were used with Boolean operators to combine searches: (“oral cancers” OR “cancers of head and neck” OR “cancer of tongue” OR “cancer of oropharynx” OR “cancer of hypopharynx” OR “cancer of esophagus OR cancers of the UADT” AND “dental visits” OR “visits the dentist” OR “dental check-up” OR “dental examination” OR “dental treatment” OR “dental care” OR “oral hygiene” OR “periodontitis”) with no limitations on year of publication and language (Table 1). A health librarian reviewed and provided input on the search strategy. Additional search strategies included (i) a hand search of the reference lists of included studies, (ii) the use of the “related citations” feature in PubMed, (iii) an ongoing surveillance of the literature while updating the manuscript, and (iv) authors were contacted for the articles for which full text was not available. EndNote software was used to remove the duplicates for the same type of article in more than one database. Alerts with search strategies were created in the databases to maintain an ongoing surveillance of the literature.

### Study inclusion and exclusion criteria

To be eligible for inclusion, the paper had to report a primary study with any population, one or both genders specified, participants of any age, incidence of any cancer subsite of HN, and UADT reported as the health outcome, frequency of dental visits/check-ups prior to the diagnosis of one of these cancers (assessed as the exposure), and availability of sufficient data to estimate the measure of association, i.e., unadjusted odds ratio (OR) along with its corresponding 95% confidence interval (CI). Where a single study was described in several publications, the study which reported the incidence data most comprehensively was included in the analysis. Gray literature, such as unindexed or unpublished conference proceedings, pre-prints, and state of art reports, were not included due to limited resources to access the same.

### Data extraction

Two reviewers (B.G. and N.K.) independently screened the title and abstract of the identified citations. Full texts of citations judged as potentially eligible were acquired by at least one of the two reviewers. Thereafter, both the reviewers used a standardized and pilot-tested form to independently screen every full text for eligibility. Disagreements were resolved by consensus among the authors. Data extraction from individual studies included information on first author’s last name, year of publication, region of study, study design, number of cases and controls (or number of participants and events), population characteristics (gender and age), exposure definition (frequency or reason of dental visits which were defined as never/only when in pain, every 6 months or less and every 6–12 months, less than once a year and more than once a year, 1–2 in a year, 3–5 in a year and > 5 in a year, ≥ once every 5 years versus < once every 5 years, never versus yes, no regular/special dental care versus regular/special dental care), definition of cancer site, its subsite and its diagnostic/confirmation criteria, method of selection of controls (hospital/population based), adjusted covariates in the regression model, and risk measure in each reviewed article.

### Quality assessment

Studies were assessed for methodological quality using the quality assessment tool for quantitative studies developed by the Effective Public Health Practice Project (EPHPP) [16]. This tool has six components (selection bias, study design, confounders, blinding, data collection method, withdrawals, and dropouts). Based on this criterion, a global rating as strong, moderate, or weak was assigned to each study based on the no weak rating, one weak rating, and two or more weak ratings for any of the above mentioned six components, using the criteria described in the EPHPP dictionary itself.

### Summary measure and data synthesis

A random effects model was employed in all meta-analysis procedures which produces results that generalize to a range of populations and to different study designs in addition to accounting for heterogeneity between studies. Forest-plots were used to demonstrate the effect of each study and the summary effect size. For effect size estimates, standard errors of its logarithm were calculated from the reported or estimated CIs, assuming that the effect size was log-normally distributed. The logarithms of the effect sizes and their corresponding standard errors formed the data points for random effects meta-analysis. For each analysis, heterogeneity was assessed using by Cochran’s *Q* statistic (measure of weighted square deviations), with N-1 degrees of freedom (where *N* is the number of studies), results of statistical test based on Q statistic, between studies variance (T2), and ratio of the true heterogeneity to total observed variation (*I*^2^). We conducted sensitivity analysis by dropping one study at a time and examining its influence on the summary effect estimates. To investigate publication bias, funnel plots were constructed, plotting the logarithmically transformed ORs against the standard error of the associated log OR. The distribution of study risk estimates across the funnel plot was examined visually and Egger’s test for small study effects was performed to assess the degree of asymmetry. Comprehensive meta-analysis software was used for all analyses [17]. Unadjusted effect estimates were used in the meta-analysis as the confounding variables used in the multivariate regression model varied significantly between studies. However, unadjusted OR could not be computed for two studies due to limited data on number of cases and controls [18, 19].

The classification of exposure variable (history and frequency of past dental visits prior to the diagnosis of cancer for cases and disease/date of interview for the hospital or the population based controls) differed between studies, with few studies reporting more than two categories. In the second situation, a single effect estimate was computed by comparing the frequency of dental visits in highest category versus the pooled data from the other categories.

Studies that evaluated cancers of the HN/UADT and oral cavity were pooled in two separate evaluations. Some studies presented data on both HNCs as well as OCs and were included in both evaluations. Two different meta-analyses were conducted based on the health outcome as reported by the authors in the articles: one for HNCs/UADTCs and the other meta-analysis was conducted based on OCs exclusively. To report the pooled effect of frequency of dental visits on incidence of cancers, the exposure variable was categorized as yes/regular/frequent (subjects in highest category of dental visits in each study) and never/irregular/not frequent (other dental visits categories of each study). A third meta-analysis was executed by pooling the data from all the studies irrespective of the cancers with the frequency of dental visits as subgroup. Three studies could not be included in any of the other subgroups and shared individual identity for subgroups.

## Results

**Fig. 1:**
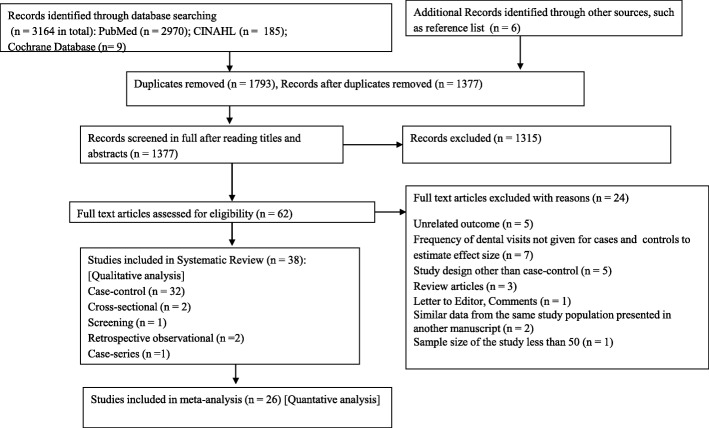
Flowchart diagram of literature search according to Preferred Reporting Items for Systematic Review and Meta-Analysis guidelines

The detailed process of the literature search and article screening is described in Fig. 1. The database and the hand search of the reference list yielded 3170 publications. The databases used as sources for studies were PubMed (*n* = 2970), CINAHL (*n* = 185), and Cochrane Library (*n* = 9). A total of 1377 articles remained after excluding 1793 duplicate records. A further 1315 articles were excluded after study of the abstracts, leaving 62 for which the full texts were assessed for eligibility. The systematic review finally included 38 articles after excluding 24 articles due to unrelated outcome, frequency of dental visits not given for cases and controls to estimate the effect size, study design other than case-control, review articles, letter to editor or comments, similar data from same study population presented in another manuscript, and sample size less than 50. Finally, a total of 26 case-control studies were included in the meta-analysis. The summary and the characteristics of these articles are presented in Tables 2 and 3.

### Characteristics of included articles

Overall, this systematic review includes 32 case-control and 6 other design studies not limited to cross-sectional, observational, case-series, and screening. Fourteen of these studies were conducted in Europe [20–33]. There were nine studies conducted in North America [1, 2, 4, 18, 34–38], five in South America [39–43] nine in Asia [44–52], and a single study in Australia [53]. For the meta-analysis, 16 case-control studies had hospital-based controls [2, 20, 23, 27, 28, 30, 36, 39, 41, 43–45, 48, 50, 52, 54, 55], seven had population-based controls [18, 24, 29, 34, 35, 47, 56], and two studies had both hospital- and population-based controls [22, 40]. There was one study on OCs conducted in India, and this did not provide the source, nor describe the type of controls [49].

Among the studies included in the meta-analysis, 11 were based on HNCs and 13 studies included OCs as the health outcome (details of same available in Tables 2 and 3). Only two studies included both HNCs and cancers of the esophagus [22, 24]. The studies with HNCs/UADTCs represented a total sample size of 38,552 including 17,313 cases and 21,239 controls. The studies with OCs represented a total sample size of 22,542 including 10,982 cases and 22,542 controls. All the studies included both males and females with one exception which included only females, this being conducted in China with OCs as the health outcome [44]. The overall age group of the study participants ranged from < 20 to ≥ 80 years. Dental visits as never/only when in pain or less than once a year and more than once a year were reported in 11 studies. One study reported frequency of dental visits as 1–2 in a year, 3–5 in a year, and > 5 in a year. Never, only when in pain, and regular dental visits were reported in one study. Never, every 6 months or less, and every 6–12 months dental visits were reported in three studies. Dental visits as never, < every 5 years, every 2–5 years, and at least every year were reported in three studies. Never, ≥ once every 5 years versus < once every 5 years dental visits were reported in two studies. Dental visits as never versus yes were reported in four studies. No regular/special dental care versus regular/special dental care was used in one study.

### Quality assessment

Majority of the studies (29) were of strong quality. Moderate quality was assessed for seven studies and three studies were assessed as weak (Table 4).

### Meta-analysis for HNCs/UADTCs

**Fig. 2:**
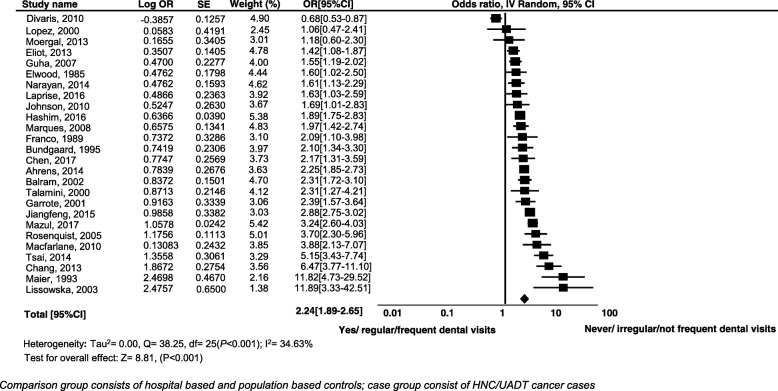
Forest plot of effect of history of dental visits and incidence of HNCs/UADTCs

The odds of never/irregular/not frequent dental visits as compared to yes/regular/frequent dental visits were greater among the cases as compared to controls. Never/irregular/not frequent dental visits increased the risk of cancer significantly. Under the random effects model, the overall pooled estimate risk for cancer was (OR 2.24; 95% CI 1.89, 2.65, *P* < 0.001). The test for heterogeneity produced Tau square of 0.00, *Q* = 38.25, *I*^2^ = 34.63%, test for overall effect *z* = 8.81, (*P* < 0.001). The highest risk estimates observed were (OR 11.89; 95% CI 3.33, 42.51, *P* < 0.001) in a study conducted in Poland from 1997 to 2000. However, the wide CIs indicate the small sample size of the study. We did not find any statistically significant results for three studies (Fig. 2).

### Meta-analysis for OCs

**Fig. 3:**
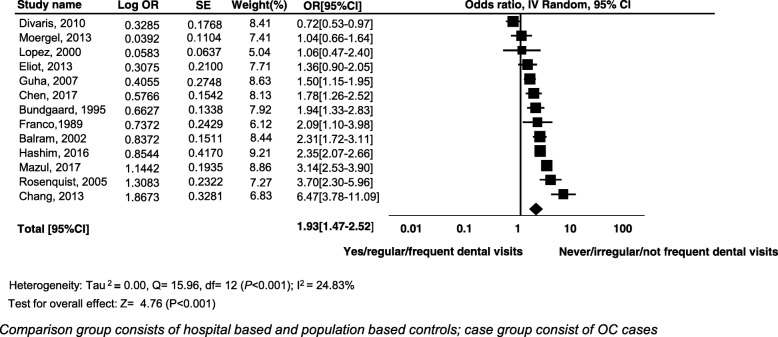
Forest plot of effect of history of dental visits and incidence of cancers of the oral cavity

The odds of never/irregular/not frequent dental visits as compared to yes/regular/frequent dental visits were higher among the cases as compared to controls indicating a statistically significant increase in the cancer. Under the random effects model, the overall pooled estimate risk was (OR 1.93; 95% CI 1.47 to 2.52, *P* < 0.001). The test for heterogeneity produced Tau square of 0.00, *Q* = 15.96, *I*^2^ = 24.83%, test for overall effect *z* = 4.76, (*P* < 0.001). Highest risk was found in a study conducted in Taiwan on 287 cases and 296 controls (OR 6.47; 95% CI 3.78 to 11.09, *P* < 0.001) (Fig. 3). We also observed that yes/regular/frequent dental visits showed protective effect and decreased the incidence of OC by 52%. The overall pooled estimate risk for cancer was (OR 0.48; CI 0.38 to 0.60, *P* < 0.001), data not shown.

### Meta-analysis for HNCs/UADTCs by subgroup analysis

**Fig. 4:**
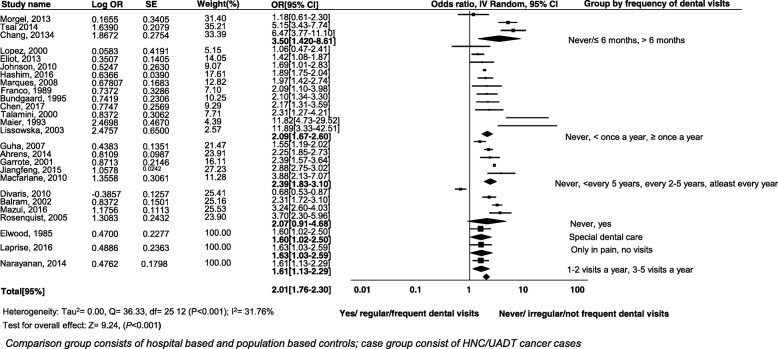
Forest plot of effect of history of dental visits as subgroup and incidence of HNCs/UADTCs

The studies were divided into various subgroups according to reported frequency of dental visits in respective studies as follows: (Never/≤ 6 months, > 6 months); (Never, < once a year, ≥ once a year); (Never, yes); (Special dental care); (only in pain, no visits); (1–2 visits a year, 3–5 visits a year). Figure 4 illustrates the subgroup analysis by frequency of past dental visits in these studies. The overall pooled estimate risk was (OR 2.01; 95% CI 1.76 to 2.30, *P* < 0.001). The test for heterogeneity produced Tau square of 0.00, *Q* = 36.33, *I*^2^ = 31.76%, test for overall effect *z* = 9.24, (*P* < 0.001).

### Publication bias and meta-regression

**Fig. 5:**
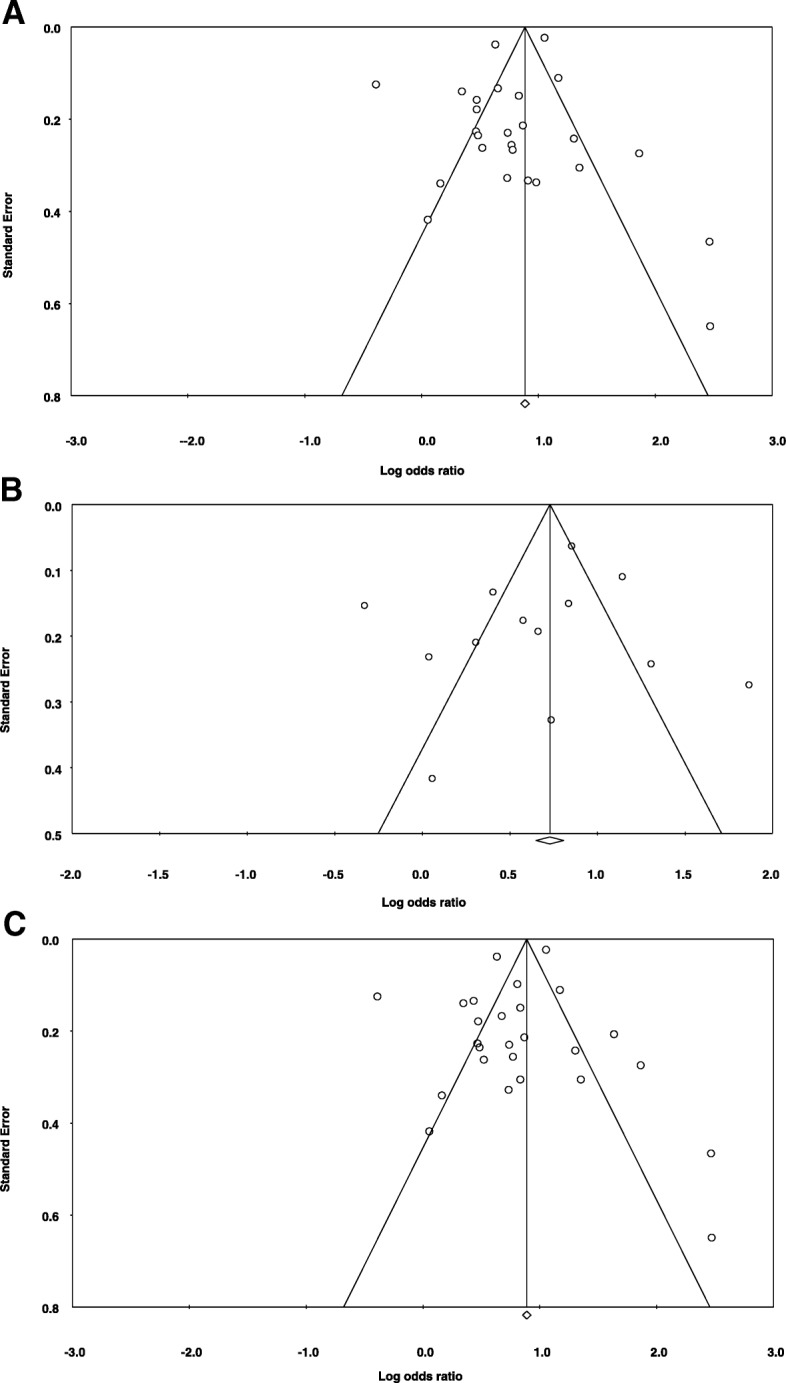
**a** Funnel plot of standard error by Log odds ratio for all HNCs/UADTCs case-control studies. **b** Funnel plot of standard error by log odds ratio for all OCs case-control studies. **c** Funnel plot of standard error by log odds ratio for all HNCs/UADTCs studies grouped by frequency of dental visits

The symmetrical funnel plot in Fig. 5a–c by visual inspection for frequency of dental visits and incidence of HNCs/UADTCs and OC indicates that there was no publication bias in our meta-analysis. The Egger’s regression intercept was − 0.9849, standard error = 0.8461, 95% CI − 2.73 to 0.76, *t* = 1.16, df = 24, and *P* = 0.2558. Publication bias for studies conducted on OCs shows Egger’s regression intercept = − 1.3239, standard error = 1.7850, 95% CI − 5.25 to 2.60, *t* = 0.7416, df = 11, and *P* = 0.474. For all the studies included in meta-analysis and grouped by the frequency of dental visits: the Egger’s regression intercept was − 0.889, standard error = 0.8946, 95% CI − 2.73 to 0.9941, *t* = 0.9940, df = 24, and *P* = 0.330. Results of meta-regression analysis indicate that none of the included subgroups for history of dental visits influenced the effect estimate (Table 5).

## Discussion

In this meta-analysis, we aimed to quantify the effect of frequency of dental visits on incidence of HNCs/UADTCs and OCs. To the best of our knowledge, this is the first systematic review and meta-analysis on this topic. All the included 26 studies in meta-analysis were hospital- or population-based case-control studies, which included history of dental visits among incident cancer cases. Our meta-analysis irrespective of the frequency of past dental visits indicates a significant association between lack of dental visits (never/irregular/not frequent) and incidence of HNCs, particularly so for OCs. This may be partly attributed to the hypothesis that individuals not visiting the dentists for oral check-ups are ignorant of their oral hygiene and any potentially malignant changes in their oral cavity. Other reasons could be perceived lack of need, affordability, limited resources, and limited availability of oral health care providers and populations with low gross national income per capita.

Lack of dental visits on regular or frequent basis has been posited to contribute to the incidence of, and outcomes for HNCs, as a synergistic and independent variable [20, 40, 53]. Several studies report that subjects who had never made any dental visits had a higher risk of OCs than subjects who reported visiting at least once a year [2, 27, 37, 40, 42, 50, 51, 56, 57]. Two studies indicate significant increase in the risk of OCs in the absence of dental visits among women only [44, 52].

Results of a case-series on 441 incident cases of oral and oropharyngeal cancer in the Greater Boston area reports that never or rarely going to the dentist was associated with being diagnosed at higher stage (cumulative OR = 2.28, 95% CI 1.02 to 5.10 and cumulative OR = 9.17, 95% CI: 2.70 to 31.15) compared to those going to the dentist at least annually [4]. The Carolina head and neck cancer population-based case-control study found a 32% decrease in oral, pharyngeal, and larynx cancers among those who had a history of more than one or more routine dental visits in the past ten years from the date of diagnosis of cancer [18].

Pooled analysis of 8925 incident cases of HNCs and 12,527 controls of 13 INHANCE case-control studies found 26% reduction in incidence of OCs among the patients who made one or more than one annual visits for dental check-ups [20]. This result of substantial decrease in the risk of OCs associated with regular or routine dental visits is in concordance with a substantial body of previous epidemiological studies [24, 29, 30, 33, 37, 39–41, 43, 52, 56]. However, there are studies which do not report any significant positive association between dental visits and incidence of OCs [23, 28, 40]. Mazul et al. supported the evidence that routine dental examinations were significantly associated with decreased risk of all UADT cancer subsites [34].

We have made every effort to include all case-control studies with adequate sample size (*N* > 50) so that the statistical power and precision of analysis in this paper are strong, and ensure generalizability of our results. Furthermore, majority of the studies we have chosen are of sufficiently good quality.

Our findings have wider clinical implications. Regular or frequent dental check-ups, with oral hygiene advice and interventions, will aid in maintaining a health-associated oral flora, reducing the load of potential pathogens. Dentists can expedite early detection of OCs and move a patient quickly into available management pathways. There have been statistically significant differences in the percentage of OCs identified by the dentists and oral maxillofacial surgeons in comparison to the medical physicians in regions like Australia and Europe [32, 53, 58]. Similarly, there is an abundant evidence for dentists in contrast to the physicians making referral for OCs at an early stage [3, 53, 59–63]. Several past studies add to the referral pattern of the OCs patients made by the dental practitioners at an early stage embarking the significant role of dentists in disease detection [63–69].

Langevin et al. in a population-based case-control study of HNCs in the greater Boston area and Holmes et al. in a retrospective study in a central European population have reported that oral and oropharyngeal cancers were diagnosed at an early stage at dental offices, compared to physician’s offices [4, 62]. Concurrently, a study conducted on 131 incident cases of oral and oropharyngeal cancers in Florida showed that regular dental visits were associated with 65% of cancers being diagnosed at an early stage [1].

Routine opportunistic screening is long recommended for all dentists as they have access to full mouth examination during routine dental check-ups and are well aware of normal oral anatomy [70]. There is evidence for support from a retrospective cohort analysis in Canada on 2331 incident cases of squamous cell carcinoma of anterior tongue and floor of mouth that the dentists were more likely to have detected the cancer through a screening exam (15% compared to 1.4% referred by a family doctor) [3]. Nonetheless, OCs in its initial stage is not always clinically detectable and requires special skills, including palpation of suspect tissues [11, 71, 72] Continuing education programmes for dentists for early detection of OCs are advocated by many and are practiced in many countries now, frequently managed by national dental, otorhinolaryngeal, or HN oncological associations [71, 73].

### Limitations

Due to limited resources, this review searched and included studies from three large commonly used databases only. Greater availability of resources and access may have allowed a wider search yielding more studies (published and unpublished) via other databases, web resources, and gray literature. There is also a possibility of having excluded some studies which reported/published insufficient data to meet the inclusion criteria of this review when these studies may have had adequate unpublished data. However, it was beyond the scope of this review to trace this data.

Though most of the studies were of strong quality, they differed in nature of population, study setting, cancer case definition, method of diagnosis of cancer, selection and type of controls, history of frequency of dental visits, method of data collection, and its analysis. Also, the positive association between never/irregular dental visits and incidence of cancer could be understated in our pooled estimate as this meta-analysis is conducted on case-control studies where recall and selection bias remains a concern. Another potential limitation is the varied distribution of frequency of dental visits among the studies. Unfortunately, not all dentists consider it mandatory to undertake a comprehensive examination of the oral and oropharyngeal soft tissues at the time of visits made for issues involving strictly dental problems, such as toothache or the need for dental restorations.

## Conclusions and implications for future research

Oral health is a part of general health and quality of life. Targeted education to alert those at risk about OCs and other HNCs, and the warning signs, and better training coupled with opportunistic oral cavity examinations by dentists could reduce the burden of this disease. According to The American Cancer Society, a cancer-related check-up annually for all individuals aged 40 and older, and every 3 years for those between the ages of 20 and 39, should include health counseling and examinations for cancers of the oral cavity [74]. Despite the lack of support for population-based screening, opportunistic screening by thorough examination of the oral cavity and oropharynx should be carried out while treating or examining patients for other diseases, such as caries and periodontal disease. Among other ways, a safety net could be introduced by means of compulsory dental check-ups for disadvantaged people, for example those claiming social benefits.

### Glossary of terms

Please refer to Table 6.

## Appendix

**Table 1:** Electronic database search strategy (August 2017)

PubMed	
# 1: Head and neck cancers [MH] OR head and neck neoplasms [tiab] OR oral cancers [MH] OR upper aerodigestive tract cancers [MH] OR cancer of tongue [tiab] OR cancer of oropharynx [tiab] OR cancer of hypopharynx [tiab] # 2: Dental visits [MH] OR visits the dentist [tiab] OR dental check-ups [tiab] OR dental examination [tiab] OR dental treatment [tiab] OR dental care [tiab] OR oral hygiene [tiab] OR periodontitis [tiab]	
#1 and #2	

**Table 2:** Background characteristics and data collection of the included case-control studies

Reference	Region	Time frame of study	Design of study	Gender	Age, years [mean (standard deviation) or range]	Health outcome as defined in the manuscript	Definition of cancer cases	No of cases	No of controls	Total	Definition of dental visits	Adjusted covariates	Quality assessment
Chen et al. [44]	China	2010–2015	CCH	F	20–80	OCs (not defined)	Incident cases confirmed by histology	250	996	1246	Never, < once a year, ≥ once a year	Age, marital status, residence, family history of cancer, passive smoking, exposure to cooking oil fumes, and diet containing vegetablesand fruit	Strong
Mazul et al. [34]	North Carolina	2002–2006	CCP	M + F	20–80	HNCs (oral cavity, pharynx or larynx)	Diagnosed cases	491	1396	1887	Yes or no	Age, gender & race	Strong
Hashim et al. [20]	United States, Central Europe, Latin America, Japan & Asia	2001–2009	CCH	M + F	< 40–≤ 75	HNCs (oral cavity, larynx, oropharynx, and hypopharynx)	Incident cases	3551	2748	6299	≥ Once a year versus < than once a year	Age, center, sex, education, alcohol consumption, and tobacco smoking	Strong
Laprise et al. [45]	India (Kerala)	2008–2012	CCH	M + F	Mcases = 60.1 (10.8), Mcontrols = 59.2 (11.3), Fcases 59.8 (11.5), Fcontrols = 59.9 (12.1)	OCs (lip, tongue, gum, mouth, and palate)	Incident cases	350	371	721	Never, only when in pain, regularly	NA	Strong
Friemel et al. [21]	Germany	2002–2005	CCP	M + F	32–77	HNCs (tongue, gum, mouth, palate, tonsils, pharynx, and larynx	Incident cases diagnosed by pathology	276	None	276	At least once a year, every 2–5 years, less than every 5 years, never	NA	Strong
Dholam and Chouksey [46]	India	NG	CCH	M + F	18–45	OCs (lip, buccal mucosa, lower alveolus, retromolar trigone, oral tongue, floorof mouth, upper alveolus, and hard palate) and oropharynx	Incident cases	85	85	170	Every six months, once a year, less than once a year	NA	Strong
Huang J et al.[47]	China	2010–2015	CCP	M + F	> 20	OCs (not defined)	Incident cases diagnosed by pathology	414	870	1284	Never, < 5 years, ≥ 5 years	Age, gender, body mass index, occupation, education, and place of residence	Strong
Tsai et al. [48]	Taiwan	2010–2013	CCH	M + F	20–80	HNCs (oral cavity, oropharynx, hypopharynx, and larynx)	Incident cases diagnosed by pathology	436	514	950	No, every 6 months or less, every 6–12 months	Age, sex, education, cigarette smoking (pack-year categories), betel-quid chewing (pack-year categories) and alcohol drinking (frequency)	Strong
Ahrens et al.[22]	European countries: Prague, Bremen, Athens, Aviani\o, Padova, Turin, Dublin, Oslo, Glassgow, Manchester, New castke, Barcelona, Zagreb	2002–2005	CCPH	M + F	Cases = 59.8(10.1), controls = 59.8(11.8)	UADTCs (oral cavity, oropharynx, hypopharynx, larynx, or esophagus)	Incident cases	1963	1933	3896	Never, at least once a year, 2–5 years, less than every 5 years	Age, sex, study center, smoking status, cumulative tobacco consumption, cumulative alcohol consumption, professional education, consumption of fruits and vegetables	Strong
Narayan et al.[49]	India	NG	CC	M + F	21–≤ 80	OCs (buccal mucosa, tongue, gingivo-buccal sulcus, and retromolar area)	Incident cases diagnosed by histopathology	242	254	496	1–2 visits a year, 3–5 visits a year, > 5 visits a year	NG	Weak
Moergal et al.[23]	Rhineland-Palate, Germany	2011–2012	CCH	M + F	37–88	OCs (proximal to gingiva and mandibular/maxillary alveolar mucosa), floor of the mouth, tongue alveolar bone of maxilla and mandible, palate, cheek, and other locations of the mouth)	Incident cases identified from medical records	178	123	301	≤ 6 months versus > 6 months	NG	Moderate
Eliot et al.[35]	Boston, United States	2006–2011	CCP	M + F	≤ 18	HNCs (oral cavity, pharynx and larynx)	Incident cases	513	567	1080	Less than every year, at least once a year	Age, race, sex, pack-years smoked, average alcoholic drinks per week, education status, and income level	Strong
Chang et al. [50]	Taiwan	2010–2012	CCH	M + F	20–80	HNCs (cavity, oropharynx, hypopharynx, and larynx)	Incident cases diagnosed by histopathology	317	296	613	Every 6 months or less, every 6–12 months, no	Age, sex, education, cigarette smoking (pack-year categories) and betel quid chewing (pack-year categories), and alcohol drinking (frequency)	Strong
Macfarlane et al. [24]	Europe	2002–2005	CCP	M + F	< 50	UADTCs (lip, tongue, gum, mouth, and palate), pharynx, larynx, and esophagus	Incident cases	356	419	775	Never, < every 5 years, every 2–5 years, at least every year	Age, gender, education, center, smoking, and alcohol consumption	Strong
Johnson et al.[36]	Eastern Ontario region, Canada	2004–2005	CCH	M + F	≥ 35	HNCs (oral cavity, larynx, hypopharynx, and oropharynx)	Incident cases and patients diagnosed with cancer within 2 years of the date of interview	162	2679	2841	At least every 12 months, less than once a year, rarely, or never	Age, gender, education, immigrant status, and smoking	Strong
Divaris et al. [18]	46 counties of North Carolina	2002–2006	CCP	M + F	26–80	HNCs (oral, pharyngeal, and laryngeal)	Incident cases	1289	1361	2650	Yes or No	Age, sex, race, education, smoking status intensity, drinking status, cumulative ethanol consumption, fruit and vegetable consumption	Strong
Marques et al.[39]	São Paulo, southeastern Brazil	1998–2002	CCH	M + F	< 40–≥ 70	OCs (lip, tongue, gum, mouth, and palate) and pharynx (tonsil and oropharynx)	Incident cases diagnosed by histopathology	309	468	777	Regular (annually) occasional (interval between visits ≥ 2 years), never	Age, sex, schooling, smoking, alcohol consumption, and all other oral health/hygiene variables	Strong
Guha et al. [40]	Latin America	1998–2003	CCPH	M + F	< 40–≥ 70	HNCs (oral cavity, pharynx and larynx)	Incident cases confirmed by histology or cytology	2423	1824	4247	Every year, every 2–5 years, less than every 5 years, never	Age, sex, center, education, tobacco pack-years, cumulative alcohol consumption, and all other oral health variables	Strong
Rosenquist et al. [25]	Southern health care region of Sweden	2000–2004	CCP	M + F	33–89	OCs (tongue, floor of mouth) and oropharynx	Cancer cases identified from ear nose and throat department	165	320	485	Regular versus no	Tobacco and alcohol consumption	Strong
Guneri et al. [26]	Turkey	1998–2002	CCPH	M + F	Mean for cases = 56.26, for controls = 53.39	OCs (lip, tongue, floor of the mouth and gingiva, buccal mucosa, hard and soft palate)	Incident cases identified from ear nose and throat department	79	61	140	Frequent, not frequent	NG	Moderate
Lissowska et al. [27]	Warsaw, Poland	1997–2000	CCH	M + F	23–80	OCs (tongue, gum, and mouth) and oropharynx	Incident cases diagnosed by histology	122	124	246	Never versus visits at least once a year	Age, gender, place of residence, smoking, and drinking habits	Strong
Balram et al.[52]	Southern India (Bangalore, Madras and Trivandarum)	1996–1999	CCH	M + F	22–85	OCs (not defined)	Incident cases identified from 3 South Indian centers	591	582	1173	Never versus yes	Age, center, education, smoking, and drinking habits for men only	Strong
Garrote et al. [41]	Cuba	1996–1999	CCH	M + F	28–91	OCs (mouth) and oropharynx	Incident cases identified from National institute	200	200	400	Never, ≥ once every five years, < once every 5 years	Age, gender, area of residence, education, smoking, and drinking habits	Strong
Winn et al.[42]	Puerto Rico	1992–1995	CCP	M + F	21–79	OCs (tongue, gum, mouth) and pharynx (oropharynx and hypopharynx)	Incident cases diagnosed by histology	342	521	863	Yes, no, never	NG	Strong
Moreno-Lopez et al.[54]	Spain	Not mentioned	CCH	M + F	19–85	OCs (labial mucosa, tongue, gingiva, mouth) and oropharynx	Hospital diagnosed cases	75	150	225	Never, not regularly (at least once a year), regularly	NG	Strong
Talamini et al.[28]	Italy	1996–1999	CCH	M + F	27–86	OCs (tongue, mouth,) and oropharynx	Incident cases	132	148	280	Never, < once a year, ≥ once a year	Age, gender, fruit and vegetable intake, and smoking &drinking habits	Strong
Bundgaard et al.[29]	Denmark	1986–1990	CCP	M + F	≤ 45–> 70	OCs (retromolar area, buccal mucosa, floor of mouth, hard palate, upper and lower alveolus, and tongue)	Incident cases	161	483	644	At least once a year (regularly), more than once year	Tobacco and alcohol	Strong
Maier et al. [30]	Germany	1986–1989	CCH	M + F	30–75	HNCs (oral-cavity, oropharynx, hypopharynx and larynx)	Cases examined at department of maxillo-facial and head and neck surgery	100	214	314	Only in pain, less than once a year, more than once a year	NG	Moderate
Marshall et al.[37]	New York	1975–1983	CCP	M + F	≤ 50–≥ 76	HNCs (tongue, oropharynx, floor of mouth, pharynx, or hypopharynx)	Cases diagnosed pathologically	290	290	580	White patches, infection or inflammation, sharp or jagged teeth, toothache or crooked teeth	Tobacco and alcohol	Moderate
Zheng et al.[51]	China	1989–1990	CCH	M + F	18–80	OCs (tongue and mouth)	Incident cases diagnosed by histology	404	404	808	Routine visits or because of oral ulceration and toothache	NG	Strong
Franco et al.[43]	Brazil	1986–1988	CCH	M + F	< 40–≥ 70	OCs (tongue, gum, and mouth)	Incident cases diagnosed by histopathology	232	464	696	Never, < once a year, ≥ once a year	Age, sex, study site, and admission period	Strong
Elwood et al. [2]	Canada	1977–1980	CCH	M + F	20–94	HNCs (tongue, mouth, oropharynx, hypo-pharynx, and larynx)	Incident cases	374	374	748	No regular dental care versus no special dental care	Socioeconomic status, marital status, alcohol, and cigarette consumption	Strong

*CCH* case-control with hospital based controls**,** *CCP* case-control with population based controls**,** *CCPH* case-control with hospital- and population-based controls, *M* males, *F* females, *M* + *F* males and females, *NG* not given, *OCs* oral cancers, *HNCs* head and neck cancers, *UADTCs* upper aerodigestive tract cancers

**Table 3:** Background characteristics and data collection from the other design studies

Reference	Region	Time frame of study	Design of study	Gender	Age, years (average, mean with standard deviation or range)	Cancer outcome with subsite	Case definition	No of Cases	Sample population	Frequency of dental visits	Quality assessment
Bertl et al. [31]	Austria	2013	Cross-sectional	M + F	> 18	HNCs (oral cavity, nasopharynx, oropharynx, hypopharynx, or larynx)	Patients from outpatient cancer clinics	48	Tertiary hospital	< 12 months or ≥ 12 months	Moderate
Langevin et al. [4]	Greater-Boston	2006–2011	Case-series	M + F	≥ 18	OCs (tongue, salivary gland, gum, mouth), oropharynx and hypopharynx	Incident cases	441	Teaching hospital	At least annual, infrequent, rare or never	Strong
Frydrych et al.[53]	Western Australia	2005–2009	Retrospective observational study of medical records	M + F	Average age for M = 59 and for F = 60	OCs (lip, tongue, gum, mouth, palate) tonsil and oropharynx	Incident cases	127	Teaching hospital	Time (months) since the patient’s last dental visit and regularity of attending dental appointments	Moderate
Watson et al. [1]	Florida	NG	Cross-sectional	M + F	60 (13.2)	OCs (lip, oral cavity) or oropharynx	Incident cases	131	Two tertiary care centres	Within preceding 12 months, more than 12 months ago	Moderate
Lockhart et al. [38]	United States	NG	Screening	M + F	17–86	HNCs (not defined)	NG	131	HNC clinic	Emergency dental care, annually, regularly or twice yearly	Weak
Gellrich et al. [32]	Europe	NG	Retrospective observational study of medical records	M + F	Average age of M = 58.2, F = 62.6	OCs (floor of the mouth, tongue, gingiva, non-specified sites for oral cancer, excluding lip)	Incident cases	1543	Oral and maxillofacial surgery departments	Quarterly, annually, twice a year, every 2 years, less than every 2 years	Weak

**Table 4:** Quality rating of the included studies according to Effective Public Health Practice Project’s Qualitative Assessment Tool for Quantitative Studies

Reference	Overall quality assessment	Selection bias	Study design	Confounders	Blinding	Data collection	Withdrawals and drop outs
Chen et al. [44]	Strong	1	2	1	2	1	1
Mazul et al. [34]	Strong	1	2	1	2	1	2
Hashim et al. [20]	Strong	1	2	1	2	1	2
Laprise et al. [45]	Strong	2	2	1	2	1	1
Friemel et al. [21]	Strong	2	2	1	2	1	2
Dholam and Chouksey [46]	Strong	1	2	2	2	1	2
Bertl et al. [31]	Moderate	2	3	2	2	2	2
Huang J et al. [47]	Strong	1	2	1	2	1	2
Tsai et al. [48]	Strong	2	2	1	2	1	2
Ahrens et al. [22]	Strong	2	2	1	2	1	2
Narayan et al. [49]	Weak	3	3	3	2	1	3
Moergal et al. [23]	Moderate	3	2	2	2	1	2
Eliot et al. [35]	Strong	1	2	1	1	1	2
Chang et al. [50]	Strong	2	2	1	2	1	2
Langevin et al. [4]	Strong	1	2	1	2	1	2
Frydrych et al. [53]	Moderate	3	2	2	2	2	2
Groome et al. [3]	Strong	2	1	1	2	2	2
Macfarlane et al. [24]	Strong	2	2	1	2	1	2
Johnson et al. [36]	Strong	1	2	1	2	1	2
Divaris et al. [18]	Strong	2	2	1	2	1	2
Watson et al. [1]	Moderate	3	2	2	2	2	2
Marques et al. [39]	Strong	2	2	1	2	1	2
Guha et al. [40]	Strong	2	2	1	2	1	2
Rosenquist et al. [56]	Strong	1	2	1	2	1	2
Guneri et al. [26]	Moderate	1	2	2	2	1	2
Lissowska et al. [27]	Strong	1	2	1	2	1	2
Gellrich et al. [32]	Weak	1	3	1	3	1	1
Balram et al. [52]	Strong	1	2	1	2	1	2
Garrote et al. [41]	Strong	1	2	1	2	1	1
Winn et al. [42]	Strong	1	2	1	2	1	2
Moreno-Lopez et al. [54]	Strong	2	2	1	2	1	2
Talamini et al. [28]	Strong	1	2	1	2	1	2
Bundgaard et al. [29]	Strong	1	2	1	2	1	1
Lockhart et al. [38]	Weak	3	3	2	2	2	2
Maier et al. [30]	Moderate	3	2	2	2	1	2
Marshall et al. [37]	Moderate	3	2	1	2	1	2
Zheng et al. [51]	Strong	1	2	1	2	1	1
Franco et al. [43]	Strong	1	2	1	2	1	2
Elwood et al. [2]	Strong	2	2	1	2	1	1

**Table 5:** Meta-regression analysis

Moderator	Coefficient	Standard error	95% CI	*P* value
Never/≤ 6 months, > 6 months	Reference			
Never, < once a year, ≥ once a year	− 0.4954	0.3363	− 1.1545–0.1637	0.1407
Never, < every 5 years, every 2–5 years, at least every year	− 0.4048	0.3632	− 1.1167–0.3072	0.2651
Never, yes	− 0.5807	0.3370	− 1.3195–0.1581	0.1234
Special dental care	− 0.8194	0.5741	− 1.9447–0.3059	0.1535
Only in pain, no visits	− 0.8008	0.5776	− 1.9329–0.3312	0.1656
1–2 visits a year, 3–5 visits a year	− 0.8132	0.5569	− 1.9046–0.2782	0.1442

*R*^2^ analog = 0.00, test of the model: *P* = 0.6316

**Table 6:** Glossary of terms

CIs	Confidence intervals
EPHPP	Effective Public Health Practice Project
HNCs	Head and neck cancers
HN	Head and neck
ICD	International classification of disease
MOOSE	Meta-analysis of Observational Studies in Epidemiology
N	Number of studies
OCs	Oral cancers
OR	Odds ratio
PRISMA	Preferred Reporting Items for Systematic Review and Meta-Analysis
UADT	Upper aerodigestive tract
